# Intelligence of Action

**Published:** 1907-10

**Authors:** C. A. F. Lindorme

**Affiliations:** Atlanta, Ga.


### INTELLIGENCE OF ACTION.

By C. A. F. Lindorme, Ph. D., M. D., Atlanta, Ga.

Audubon, one evening, in his boat on the Ohio river, watching a goshawk chasing a flock of blackbirds, noticed a remarkable stratagem of the bird of prey. The sport left him little chance. One of the banks of the river was thickly studded with dense forest, and the small animals went for cover in the thick foliage.

If the goshawk had followed the usual method of his kind, and devoured his prey, one by one, in proportion as he caught the birds, he would have hardly had the time necessary to dispose of many more than a couple, or three, before the whole flock would have been lost to his victimizing pursuit.

What tactics did he use to get on the better side of good luck? Having come up with his game, he ran amuck in the flock, killing to the right, killing to the left, never delaying for an instant his murderous assault, but slaughtering as fast as he could, drop-

*p*ing instantaneously every bird he had killed into the river, and rushing in the thickest throng again for more victims immediately after. Subsequently, when the flock of blackbirds had disappeared in the protecting foliage of the stocked forest on the bank of the river, he leisurely returned to the field of carnage, picked up his trophies, and made a sumptuous repast, where otherwise, without the stratagem, he would have had to take his chances, mightily slim at the protracted meal-hour, and eventually he would have had to go hungry to sleep.

Now for the psychology in the case: Was it all intelligence?

We doubt the correctness of the supposition. There was more of a stomach than of brains in the business. There was soul in the hawk. But it was the spirit of rapaciousness. It was all action, no reflection. There was no thought. The plan the hawk made manifest was not a mapped-out theory, but intelligence of action. The intelligence of the hawk was a reaction of his will. His optic nerve, and in physiological order, his pneumo-gastric nerve, was powerfully impressed by the sight of the flock of blackbirds, and he improved his opportunity. But he did not ponder on the circumstances. He took them in at a glance. That made him to devise his stratagem. His measure was not the result of comparing figures, abstractedly. To do this he would have needed a language. It was a psychological outcome, but physiologically prompted. The option left to him was the alternative either to indulge at once in his gormandizing, piece-meal, a scanty prospect, or to suspend his feasting, and devote himself at first to a carnage which secured him a substantial victimizing all around.

His intuition pointed out his advantage: As a matter of fact he abstained from an immediately pleasureable indulgence, and this was a psychological move; it was an inhibitory nerve-influence, quite a feat for a goshawk; it seems to point to reflection, to thought. But there was no such thing. The fear to lose his chance made him desperate. That was the physiological prompter: he had the inventiveness of necessity. Intellectually he was in a measure outwitted by the blackbirds, when they went to cover.

But the circumstances favored him as long as the flock had not reached their point of refuge.

As to the dropping of the killed birds into the river, there is the possibility that the hawk simply ran the risk of losing his game, without giving to the eventuality further attention. But an experience in his sporting practice, that the river floated light, especially feathery objects, was by no manner of means excluded.

Physiologically and psychologically, we may say, the occurrence was, to begin with, an outward impression of the hawk, which through the par-vagus worked on his blood, excited by hunger, and caused here, under the pressure of circumstances, the impetus which determined his tactics.

It would not be the first instance that an emergency-case had led to a discovery. The old surgeons some centuries ago, used hot oil for the dressing of wounds. Ambroise Pare, an army-surgeon, in a very bloody battle, ran short of it. For want of another substitute, he resorted to spring-water.

He never, in all his life, had had more brilliant results.

What do the intellectualizing believers in their keeping action behind in the procession, and reflection ot the front of doing, think of this stroke of intelligence?

Was the achievement of knowledge in the greatest surgeon of his time more high-toned-academical than the over-reaching of the goshawk?

There is an anecdote of dazzling surgery going the rounds of the college-pulpits which is in point here. There was once a woman, I think she is said to have been the wife of a herdsman, who lay three days and three nights, in throes in child-bed. There was absolutely no progress. The pains she suffered were more than hellish, and she could not bear any longer the torture she experienced by the vain efforts of nature to free her from her burden. Art, however, at that time, did not understand either to come to the rescue with relieving measures, and there she was left powerless, with torment as terrible as any that could by legal procedure, be invented for the delinquent on the rack, in lawful prosecution of "justice."

This made her desperate. She espied a moment when her attendants had stepped out of the room, seized a razor, and made an up-and-down cut right through the walls of the abdomen and those of the uterus. long enough to procure an outlet for the fruit of her womb, and yet not so deep as to injure the child, and freed herself from the agonizing pain.

By way of this audacious step the heroic woman saved herself and her young one; the delivery proceeded in due order; not only the child lived, but the mother survived too, and the wound of the latter healed without any untoward event.

And the surgeons famlliarized themselves with the operation, which, in commemoration of the unique precedence, they might well have given a fitter denomination than that of the Cesarian section.

Now, go for the psychology of the occurrence. Was the woman perhaps a secret graduate from an obstetric institute? At the time of the dramatic happening there were even no obstetrical institutes. The woman in question was altogether unlearned, I mean, without book-learning. Then, what was it thot she had been guided by?

Absolutely nothing but the complex circumstances she was put in, and the character she owned in meeting them. The insupportable torture she was forced to undergo, by the non-delivery, gave her the intrepidity she exhibited, and what she had of surgical knowledge, if knowledge it may be called, was all in the resolution of ending the tragedy. She did not, at first, to begin with, think of the matter, technically; she did not, like many a student mid-wife, brush up, the anatomical knowledge of the parts, muscles, aponeurosis or fascia; she had no knowledge to brush-up; nor did she ponder on the length and depth of the incision she was going to make; pondering was with her altogether out of the question; when resolving the heroic deed, she did less pondering than all the time before; she was left no time for pondering; all her faculty of the action was in her doing; she did not know how thick the walls of the abdomen were, nor how thin those of the uterus, the ones as much as the others stretched into the bargain by the unwonted pelvic increment, but cut just deep enough to admit of the passage of the fetus. All her guidance was in her suffering. Yet she was far from being without all pertinent consciousness. She had the advantage of being both, party and surgeon, at the same time parturient and midwife; she felt the condition she was in, ond the intelligence, the semly miraculous skill she exhibited by her own operation upon herself, was all in the necessity of doing, the push towards going ahead in the emergency, it was the very same intelligence which the baby manifests, that when laid on, makes the sucking motion, and milks its mother, although the act ensues without preparatory lessons, least those of the alphabet, but merely on the urgency of the hope for food, and the stimulus by way of the muscular faculty of intentional pressure, which, in the infant, rendered independent from the maternal blood-circulation, waxes into self-consciousness, a consciousness not of abstract possession but of concrete existence.

And there is occasion to emphasize this point. It is ignored by many of the learned, even: Intelligence is in the will of the organism, or say drift, instead of will: it is not in the organism because it is in the individual. It is in the individual because it is in the organism.

Wonder, how could it be otherwise.

Professor Preyer, a very studious observer of childhood, with much lovingness, perspicacity and common sense, says of the infant, it learns by ever trying again. This is wonderfully true. But the brave professor forgets, that the same principle applies to the stoutest academy president in the world: it is not by thinking but by doing that we proceed; our thinking is only a rumination of our doing; the base of every achievment is action.

There are a lot of learned people in the world who do not know what is learning. They are so used to the verbal intermediary, that they forget entirely where this verbal use comes from, and what it is for. They go by rote. They get stuck the same way as financiers who take notes like gold and silver, without inquiry whether they exchange the rags for precious metal whenever they present their riff-raff of nominality.

Language is a wonderful wealth. But it must not pretend to rule, the learning, like an empty credit system, without ever exhibiting cash on call.

There has been going on considerable fuss about a probable conversation in ant-hills and bee-hives, and enthusiastic theoreticians have rushed into the arena and are sitting with their auditory nerve sharpened, listening to the supposed language of animals, to begin with the animalculi.

I have been puzzled for years myself. One summer morning, starting for my office, I noticed, all along the fence of the front yard, a curious streak of ants, four or five abreast, as on a migration. A peculiarity was, that, off and anon, one or another of the little army would break away from the uniform order, and watch the rank and file marching, and then resume his place among them.

Could these creatures talk? Could they reason?

It looked almost like it.

How could they otherwise come to an agreement?

Man, talking man, is apt to argue this way. The privilege of language by its possessor is easily overrated.

I was overawed by the question, and dared not, for a length of time, doubt the hypothesis of an animal language.

But what made me hesitate to believe it from the outset, was the circumstance, that there is nothing in man which tends so much to make him disagree as the faculty of speech. Go to a court, assuming the attribute of justice, to a political meeting, nay, to a church, and what you hear most about, is disagreement. It is the vantage ground of thought.

Now, my merry little set of emigrants were in full agreement. The procession went on in complete order, so that the watching and officering seemed to etaoinetaoin etaroin etaoin etaoinxtoO.vo and officering seemed all superfluous and gratuitous, and evidently the ruling spirit was there without communication.

There was intelligence. But it was intelligence of action, not of abstraction. They marched in good order, because it was their will, and this will was uniform.

Once, walking through a business street, I observed a negro drayman back his vehicle to the sidewalk:

"Back, back!"

He drove a very intelligent looking horse; it did as it was bidden, it backed the dray.

"Back, a little more!" said the drayman again.

The animal backed a little more.

But the driver, a jovial looking chap, was not entirely satisfied.

And the horse backed a least bit more.

"Back! A least little bit more."

"So, that will do," the darky uttered approvingly, and also the quadruped seemed to consider the job satisfactorily finished, for it weighed its head up and down, as though the motion had reference to the part its intelligence had played in the operation.

"It is astonishing," I thought.

But then the question turned up, does the horse understand English?

It looked obviously like it. And consequently, if animals can learn to understand a language they must also be able to learn to speak one.

This had to be done by experiment.

I myself owned a horse. He was a Columbian Tennessee, and a very powerful and exceptionally intelligent animal.

It was in South Florida, and my Eddy, by nature somewhat fretful anyhow, was exetremely vexed by the sand beds of most roads which abound in that country. Wherever he espied a margin which promised a more comfortable walking, he would make for it, no matter what might become of the vehicle he was hitched to.

The horse was high-spirited, and did not take the whip. A cut with the lines was sufficient to send him flying, and it was very rarely resorted to, an angry vociferation being abundant admonition.

But that made him all the fitter for an experiment.

What he usually got to hear when he was at his old tricks again was an emphatic, "What are you doing, sir?"

It was enough to make him return to where he had to be. He knew that well enough, and he behaved well until the next temptation. Then he was sure to try it again; it did not enter his head why a hard road should not be taken rather than a sandbed.

Now the problem to solve was, did he understand what was said to him in English?

The way I settled this question was this: I happened to know several languages; so, at the next turn, in place of apostrophising him in English, I used another idiom, involvnig, however, the identical utterance.

"Herrgottskreuzhimmel donnerwetter, was fallt dir ein?" being in German.

And at the next reiteration of his naughtiness,

"Sacre matin, qu est ce que tu fais?" being in French.

"Maldita sea tu alma, caballo." being in Spanish.

Beroerd paard, ben ye geck? being in Dutch, all of the admonitions being in idiomatic driver style, to come nearest the possible genius of the animal.

There was absolutely no difference. No sooner had the horse heard my voice than it obeyed the warning, no matter in what language it had been expressed, and consequently it took effect unexpressed. It was so as in the case of Mr. Parollès, when he was by his friends subjected to a mock examination:

"Boblibindo chi curmurco"

(Shakespeare: Lord, in "All's Well that Ends Well." IV,3.)

That settled the point. My horse did not understand all those foreign idioms, nor any one of them, and as little English. What it understood was the action it was in. It had the intelligence of this action in an uncommon degree, even, and this made the understanding of language unnecessary; the phonetic part of speech being all that was required for a psychological intercourse.

And this conclusion held as well with reference to the drayhorse mentioned before. The animal did not understand English. But it understood its business, and that made all understanding of language unnecessary. Supposing the horse had been spoken to by the driver out of the usual order of things, or without the accent it was used to, in conversational tone, in the midst of a drive, would it have understood?

It would have as little understood as my Columbian Tenessee understood the foreign languages he was addressed in. He did not interpret my various admonitions in various languages; it interterpreted the entire situation it found itself in, and with reference to this interpretation I should not contradict a philosopher who would say, that in ultimate analysis my theory of the intelligence of action resolved itself into energy.

There is, in the development of the infantile intellect, not a single moment when it can be said that the way of doing of nature changes. What Preyer says of the learning of the infant, holds throughout life; it is in all stages, "learning by ever trying again." If this natural actuation of faculties is disputed, it is for reason of the neglect which experiences the very first learning of infantile creatures.

One evening my daughter-in-law, with whom I board, had with her husband gone out and left her baby in charge of a very inexperienced nurse. The mother was not to stay away long and it was hoped that the little one would sleep so long. But the baby, about a year and a half old, woke up, and screamed terribly when she was not taken by her mother. The nurse tried all the tricks she knew to quiet the little one, but of no avail, and at last she came with the child to me.

I was a favorite of the infant, and she no sooner saw me than she reached after me, and was immediately still when I held her. But only for a few seconds. She started then to cry again, and turned to the nurse to be held by her. But also this shift was not satisfactory; the child was still only a few moments, and then yelled again as badly, if not worse than before, and wanted me again to hold her. I took her on my arm, and then she was still, indeed, only recommencing to cry again when she heard the voice of her mother who came home in conversation with her husband, and a final settlement of the humor did not ensue but when she got to her privileged place at the breast of her mothr.

Now, for the psychology in the case.

When the baby screamed, and kept on doing so when held by the nurse, little familiar to her, it was for reason of the absence of the mother. The child was disappointed by the strange face she saw. Therefore when she was brought to me she was still for a few moments. But I was not the right thing either. She resumed therefore her screaming and reached after the nurse again. It was not, really, to get to her though; it was simply the "trying ever again" of Professor Preyer: she saw a female figure, and so in her indistinct perception, she made for her on the impulse to get things right at last. She therefore resumed her screaming, after realizing, being held by the girl, that it was not her mother, and she turned to me again, and wanted me to hold her: not seeing her mother, as being the best she could do under the circumstances.

I watched my grandchildren with an eye to cases in point regarding the line of demarkation between physical motion and psychological exhibit and made a very instructive observation. It was a number of months later, the formerly rather delicate babe had gotten considerably on her own feet, and was very ambitious, when she saw her sister and brother do something to fall in line. So, she ran with them, when they called me to dinner, and it was extremely ludicrous to observe her standing with the other two, pouting and pointing her lips, as she saw them do it, and made a sound resembling in her imagination the words the others spoke, representing the call to dinner.

There was the margin of abstraction. But it was not there itself. The regions of mere physicality, if we may call it so, and the sphere of psychology blend, and if we fail to appreciate the concrete circumstances, which come under our observation, and not to get mixed up, this blending we have to take into account; in our abstraction we draw a straight line of division; but in nature the like is never found in the concrete cases jumps never occur; (natura non fit saltus). I hold, for instance, that, when my grandson, on an order to do some little thing, answered in the way he had heard others do, "I will do it in a minute," this is a mere copy: he kney as little what a minute was as he knew algebra; he simply caught the sound when his elders spoke, and reproduced it.

Half a millennium ago, before the invention of movable type and the printing press, books were a rarety. Copying was the way pıacticed to duplicate desired works. Writing by hand, which by the typewriter incurs now-a-days the danger to become rudimentary, was then an art fondly cultivated, and in the universities of learning the professors delivered lectures so as to enable the students to dot down verbatim. They dictated.

At present every speech of consequence, if not anticipated by publication, is followed up closely by the printing press. But the habit to dictate lectures is upheld; in all the medical colleges, without hardly any exception, it is the uniform custom to admit the students to clinics only in the latter terms, usually the very last one.
ginning. Can there be a fitter time for the exercise of the five senses than when the student is most in need of it? An experienced medical man can be talked to about a case; the mere talking will post him; he has had similar cases by the hundreds, or at least a dozen, or has come across homologous conditions sufficiently to form an idea about the description of some exceptional one. The

Now this is a turning upside down of the right thing. There is in the entire curriculum of the medical man not so urgent an indication of the necessity of a demonstration ad oculos, as in the be-
novice knows nothing. But the student is submergeu into a flood of uncouth terminology, and expected to know the terms all by heart at the next quizz.

That old system of placing a youngster with a preceptor, was not so bad after all. The trouble is, the public do not countenance it. But as to the question when clinical teaching has to begin-the answer is, as early as it is deemed advisable to develop the intelligence of action. It is in the doing of action only that we can educate masterly doing.

